# Classification of Hemiplegic Gait and Mimicked Hemiplegic Gait: A Treadmill Gait Analysis Study in Stroke Patients and Healthy Individuals

**DOI:** 10.3390/bioengineering12101074

**Published:** 2025-10-02

**Authors:** Young-ung Lee, Seungwon Kwon, Cheol-Hyun Kim, Jeong-Woo Seo, Sangkwan Lee

**Affiliations:** 1 Stroke Korean Medicine Research Center, Wonkwang University Gwangju Medical Center, Gwangju 61729, Republic of Korea; www8744@naver.com (Y.-u.L.); lambroskch@gmail.com (C.-H.K.); 2 Department of Cardiology and Neurology, Kyung Hee University College of Korean Medicine, Kyung Hee University Medical Center, Seoul 02447, Republic of Korea; kkokkottung@hanmail.net; 3 Department of Internal Medicine and Neuroscience, College of Korean Medicine, Wonkwang University, Iksan 54538, Republic of Korea; 4 Digital Health Research Division, Korea Institute of Oriental Medicine, Daejeon 34504, Republic of Korea; 5 Korean Convergence Medical Science, University of Science and Technology, Daejeon 34113, Republic of Korea

**Keywords:** stroke, hemiplegic gait, mimicked hemiplegic gait, machine learning, gait classification

## Abstract

Differentiating genuine hemiplegic gait (HG) in stroke survivors from hemiplegic-like gait voluntarily imitated by healthy adults (MHG) is essential for reliable assessment and intervention planning. Treadmill-based gait data were obtained from 79 participants—39 stroke patients (HG) and 40 healthy adults—instructed to mimic HG (MHG). Forty-eight spatiotemporal and force-related variables were extracted. Random Forest, support vector machine (SVM), and logistic regression classifiers were trained with (i) the full feature set and (ii) the 10 most important features selected via Random Forest Gini importance. Performance was assessed with 5-fold stratified cross-validation and an 80/20 hold-out test, using accuracy, F1-score, and the area under the receiver operating characteristic curve (AUC). All models achieved high discrimination (AUC > 0.93). The SVM attained perfect discrimination (AUC = 1.000, test set) with the full feature set and maintained excellent accuracy (AUC = 0.983) with only the top 10 features. Temporal asymmetries, delayed vertical ground reaction force peaks, and mediolateral spatial instability ranked highest in importance. Reduced-feature models showed negligible performance loss, highlighting their parsimony and interpretability. Supervised machine learning algorithms can accurately distinguish true hemiplegic gait from mimicked patterns using a compact subset of gait features. The findings support data-driven, time-efficient gait assessments for clinical neurorehabilitation and for validating experimental protocols that rely on gait imitation.

## 1. Introduction

Stroke refers to neurological symptoms caused by damage to brain tissue due to either the blockage of blood vessels (ischemic stroke) or their rupture (hemorrhagic stroke) [1]. It is one of the leading causes of long-term disability, with more than 80% of survivors experiencing gait disturbances [2]. Even with continuous rehabilitation, approximately 18% of stroke patients are unable to walk, 11% require assistance, and only about half can regain independent walking ability [2]. The most common gait abnormality among stroke survivors is hemiplegic gait, which results from muscular weakness, impaired motor control, proprioceptive deficits, and increased muscle tone on the affected side [3]. This typically manifests as a flexor synergy in the upper limb and an extensor synergy in the lower limb, leading to joint patterns such as hip extension and internal rotation, knee extension, and ankle plantarflexion and inversion [1]. To achieve foot clearance during swing, patients often compensate through hip hiking or circumduction [4]. These compensatory strategies alter gait mechanics, causing increased energy expenditure and fatigue, and consequently elevate the risk of falls [5]. Therefore, the recovery of gait ability is a critical objective in stroke rehabilitation to promote independent daily functioning [6]. Recent studies have focused on quantifying gait impairments and identifying key variables for recovery using advanced analytical methods, including gait analysis systems, wearable sensors, and instrumented treadmills [7]. Given the distinguishable gait characteristics of stroke survivors, researchers have applied machine learning (ML) and deep learning techniques to develop automated algorithms capable of identifying gait deviations, predicting recovery outcomes, and classifying stroke severity [8]. These data-driven approaches have demonstrated promising performance; however, existing models primarily focus on distinguishing normal and pathological gait patterns under natural conditions. In real-world clinical and administrative settings, there are cases where patients may exaggerate or mimic hemiplegic gait, either consciously or due to external incentives such as insurance claims or return-to-work assessments. A previous study documented abnormal behaviors during rehabilitation by a stroke patient involved in a traffic accident [9], raising concerns about potential malingering or fraud. Other reports suggest that caregivers may also influence evaluations, as in Munchausen Syndrome by Proxy [10]. It is particularly challenging to detect mimicked hemiplegic gait visually because it is relatively easy to reproduce spatiotemporal patterns such as foot drop or pelvic tilting. Kinematic imitation involving joint angles can often be controlled voluntarily, making it difficult for clinicians to reliably differentiate between neurologically impaired and cognitively generated gait patterns. Despite growing interest in the objective quantification of gait impairments, there remains a lack of research specifically addressing the differentiation between genuine hemiplegic gait and its mimicked counterpart. Prior machine learning studies have not explored whether such models can detect intention-based imitation, a scenario with meaningful implications in clinical diagnostics, functional evaluations, and medico-legal contexts. Moreover, the interpretability, efficiency, and reproducibility of gait classification models remain underexplored, especially in settings where visual assessment may be subjective or inconsistent. The objective of this study was to determine whether machine learning techniques could accurately classify mimicked hemiplegic gait performed by healthy individuals and actual hemiplegic gait exhibited by stroke patients. Using treadmill-based spatiotemporal and force-related gait parameters, we developed and evaluated three supervised classifiers—Random Forest, Support Vector Machine (SVM), and Logistic Regression. We also investigated whether reduced-feature models using the top 10 most important gait parameters could maintain classification performance. Ultimately, this study aims to enhance the objectivity and reliability of clinical gait assessment, particularly in ambiguous or bias-prone scenarios, and to support functional evaluations for rehabilitation planning, insurance verification, and return-to-work decisions.

## 2. Materials and Methods

### 2.1. Data Sources and Participant Selection

This study retrospectively analyzed individuals who underwent gait analysis through a treadmill gait analysis system at Wonkwang University Korean Medicine Hospital in Gwangju (WKUGH). As this research only used simple measurement or observation equipment that does not lead to physical changes, it received review approval from the institutional review board of the hospital (WKIRB 2022-07, 29 June 2022). We selected data from subjects who visited WKUGH between November 1 2018 and June 30 2022, and who met the inclusion criteria for both the hemiplegic gait (HG) group and the mimicked hemiplegic gait (MHG) group and did not fall under the exclusion criteria (Table 1).

A total of 79 subjects were retrospectively analyzed in this study, consisting of 39 in the hemiplegic gait group (19 with left hemiparesis, 20 with right hemiparesis) and 40 in the mimicked hemiplegic gait group (20 mimicking left, 20 mimicking right hemiparesis) (Table 2).

### 2.2. Experiment and Equipment

**Figure 1:**
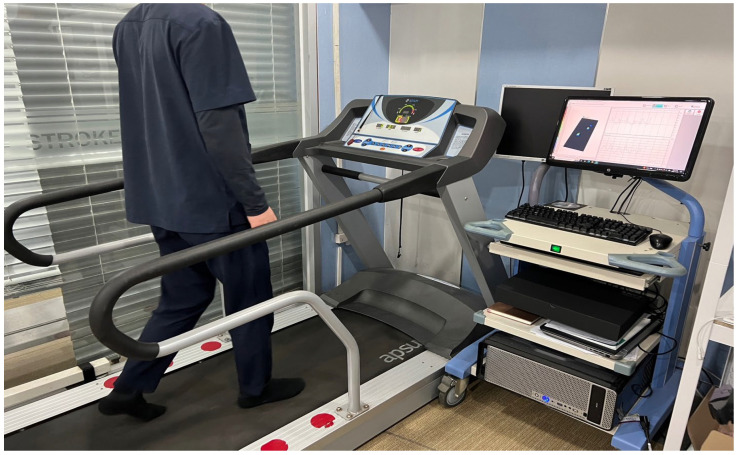
Gait analysis system using treadmill.

We conducted a gait analysis using a treadmill equipped with a pressure plate (Figure 1). When the subject starts walking on the treadmill, the pressure exerted on the pressure plate is measured, and spatiotemporal features of gait are collected. The pressure applied to the treadmill (AP2010-2Si, Apsun Inc., Seoul, Republic of Korea) is transmitted to the Zebris FDM software (version 1.18.44, Zebris Medical GmbH, Isny/Allgäu, Germany) on the computer [11].

For the HG group, the subjects were asked to walk on the treadmill at their preferred speed for 30 s. For the MHG group, we conducted training on the flexor synergy pattern of the upper limbs and the extensor synergy pattern of the lower limbs, which are characteristic of post-stroke hemiparesis. They were instructed to mimic the characteristics of hemiplegic gait, including hip extension and internal rotation, knee extension, and plantar flexion and inversion of the ankle, while performing hip hike and circumduction during swing phase. Gait analysis was conducted when it was judged that they could sufficiently reproduce the hemiplegic gait after watching videos of actual hemiplegic gait of stroke patients and demonstrations by doctors of Korean Medicine. For the MHG group, the walking speed was limited to 0.7 km/h, which is the average walking speed of the HG group, as they tended to perform normal walking when the walking speed was set high.

### 2.3. Gait Feature

**Figure 2:**
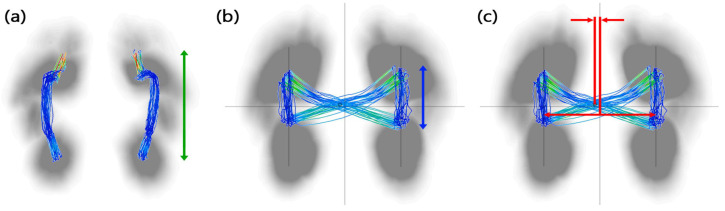
(**a**) Length of gait line (green), (**b**) single-limb support line (blue), and (**c**) lateral symmetry (red). The lines of single-limb support and lateral symmetry are derived from the butterfly-shaped diagram that illustrates the trajectory of the COP during walking.

The spatiotemporal features such as foot rotation degree, step length, stride length, velocity, cadence, and others, as well as the movement of the center of pressure (CoP) during gait, the ratio of the gait cycle of each lower limb, and the vertical ground reaction force (vGRF) generated during gait are calculated and obtained (Figure 2). These methods have been described previously [12].

The gait cycle is split into the stance and swing phases, which are determined by the toe off. The initial contact of the opposite side (left side in this case) happens when the right side reaches 50% of the cycle. There are two double limb support phases where both feet touch the ground: once at the start of the cycle (0–10%) and again midway (50–60%) (Table 3). Details of the gait cycle phases have been described previously [13].

### 2.4. Statistical Analysis

All statistical analyses were performed using Python (version 3.10) and open source scientific libraries. Data preprocessing and management were conducted using Pandas, and group comparisons between the hemiplegic gait (HG) and mimicked hemiplegic gait (MHG) groups were performed using independent *t*-tests. The *t*-tests were implemented via the SciPy package [14].

To facilitate interpretation, since the direction of hemiparesis varies among the subjects, features that can be obtained from one side of the body (foot rotation, step length, stance phase, load response, single-limb support, pre-swing, swing phase, step time, length of gait line, single-limb support line, time maximum force 1) were uniformly converted to (+) for the unaffected side and (−) for the affected side, based on the study by Lee et al. [15].

For lateral symmetry, the unaffected direction was converted to (+) and the affected direction to (−). Additionally, to interpret time maximum force 1, the time maximum force ratio was calculated by dividing the unaffected side feature by the affected side feature, referencing Patterson’s study method [16].

To assess between-group differences in gait features while controlling for the effect of age, an Analysis of Covariance (ANCOVA) was conducted for each dependent feature. The primary fixed factor was group type (hemiplegic gait [HG] vs. mimicked hemiplegic gait [MHG]), and age was treated as a covariate. The model also included the interaction term between age and group to test whether the relationship between age and the dependent feature differed across groups.

To examine group differences in gait features between the HG and MHG groups, appropriate statistical tests were selected based on normality assessments using the Shapiro–Wilk test. For features in which both groups satisfied the assumption of normality (*p* > 0.05), independent samples *t*-tests were employed. For non-normally distributed features, the non-parametric Mann–Whitney U test was applied.

### 2.5. Machine Learning Model

Since there is no established formula for calculating the required sample size in machine learning, we referred to the empirical rule-of-thumb for multiple regression proposed, which recommends a minimum sample size of N ≥ 50 + 8 m, where m is the number of predictors [17]. For a maximum of 10 predictors, this criterion suggests that at least 130 participants are needed. However, our study included only 79 participants, falling short of this recommendation.

A post hoc sensitivity analysis was conducted as follows: for the full model test with m = 10 predictors and N = 79 participants, the numerator and denominator degrees of freedom were set to df_1_ = m = 10 and df_2_ = N − m − 1 = 79 − 10 − 1 = 68, respectively. At a significance level of α = 0.05, the critical F-value was $F_{crit}$(10,68) ≈ 1.973. The non-centrality parameter λ required to achieve 80% power was obtained from the noncentral F-distribution as $\lambda^{*}$≈18.4875. Based on this, the minimum detectable effect size was calculated as $f^{2}$=$\frac{\lambda^{*}}{df_{2}}\approx 0.27$, $R^{2}=\frac{f^{2}}{1+f^{2}}\approx 0.21$. This result indicates that while the study had adequate power to detect medium-to-large effects, it may have failed to detect smaller true effects. According to Cohen’s conventional thresholds for multiple regression (f^2^ = 0.02 for small, f^2^ = 0.15 for medium, and f^2^ = 0.35 for large effects), the calculated f^2^ value of 0.2 corresponds to a medium-sized effect. This suggests that although the independent variables included in the model influence the dependent variable, the magnitude of this influence may not be substantial. Nevertheless, even very small effects can have considerable practical or clinical importance if they affect a large population.

A total of 79 observations were included in the data-set, consisting of two groups labeled as hemiplegic gait (HG) and mimicked hemiplegic gait (MHG) groups. Each observation included 26 spatiotemporal and force-related gait features, excluding age-sensitive features based on prior ANCOVA results.

To reduce model complexity and improve generalization due to the limited data-set size, feature selection was performed using the Gini impurity-based feature importance scores computed from a Random Forest classifier [18]. Specifically, a Random Forest model was trained on the entire standardized dataset using all available input features, and the importance of each feature was quantified by averaging the total reduction in Gini impurity that the feature contributed across all decision trees in the ensemble. Features that contributed more to reducing impurity were assigned higher importance scores. The features were then ranked in descending order based on their computed importance values, and the top 10 features were selected. These top-ranked features were consistently used in all subsequent modeling, hyperparameter tuning, and evaluation procedures to ensure comparability and to reduce the risk of overfitting [19].

To classify HG versus MHG by gait features, supervised machine learning models were developed using the top 10 features identified through feature selection. Three classification algorithms were employed: a Random Forest classifier, a Support Vector Machine (SVM) with a radial basis function (RBF) kernel [20], and a logistic regression model.

Random Forest is an ensemble method that constructs multiple decision trees and aggregates their outputs to improve prediction robustness. SVM identifies the optimal hyperplane to separate different classes, while Logistic Regression models the probability of class membership using a linear function. These models were chosen for their complementary strengths and their established use in biomedical data analysis

All models were implemented using scikit-learn (v1.3.0) with default hyperparameters initially, followed by hyperparameter tuning via grid search [21]. The Random Forest model leveraged ensemble decision trees to enhance robustness and interpretability, while the SVM model offered nonlinear boundary discrimination. Logistic regression was included for its simplicity and high interpretability in clinical contexts. All models were trained and evaluated on the same standardized feature set to ensure comparability across methods.

To mitigate the risk of overfitting due to the limited sample size, models were primarily evaluated using 5-fold stratified cross-validation, ensuring balanced representation of both gait groups within each fold [22]. In addition to cross-validation, an 80:20 holdout split was performed to further validate model performance on unseen data. Specifically, 80% of the data-set was randomly selected and used as a training-set for model fitting and hyperparameter tuning, while the remaining 20% was reserved as an independent test set for performance evaluation [23]. Model performance was assessed using accuracy, F1-score, and area under the Receiver Operating Characteristic—Area Under the Curve (ROC AUC), which reflect overall classification accuracy, robustness to class imbalance, and discriminative ability, respectively.

## 3. Results

### 3.1. Result of Age Effect

**Figure 3:**
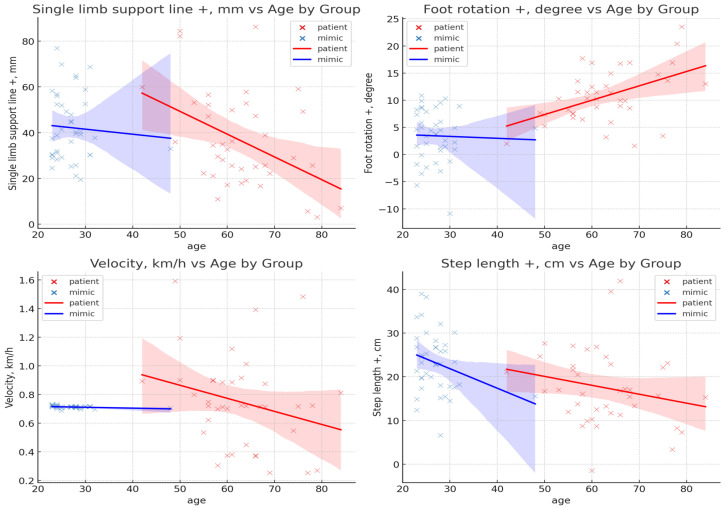
Distributional characteristics of features significantly influenced by age.

To examine the effect of age on gait features and to control for potential confounding, an analysis of covariance (ANCOVA) was performed with age as a covariate. The results indicated that age had a statistically significant effect on multiple features, including single-limb support line (+) (*p* = 0.003), foot rotation (+) (*p* = 0.007), velocity (*p* = 0.037), and step length (+) (*p* = 0.049) (Figure 3). No significant age-related effects were found in the remaining features. Additionally, no significant age × group interaction was found in any feature, suggesting that the effect of age was consistent across both groups.

### 3.2. Result of Gait Feature

To examine group differences, independent samples *t*-tests were conducted for each variable, and Cohen’s d was calculated as a measure of effect size. Furthermore, to assess the relative importance of the variables, a Random Forest classification model was employed, and the Gini importance for each variable was computed as the average decrease in Gini impurity contributed by that variable across the ensemble. The results revealed that several features showed statistically significant differences between the HG and MHG groups. Specifically, swing phase (−) (*p* < 0.001, Cohen’s d = −2.103), stance phase (−) (*p* < 0.001, d = 2.103), and time maximum force1 (−) (*p* < 0.001, d = 1.556) showed large to very large effect sizes, indicating substantial biomechanical differences. Additionally, Time maximum force ratio (*p* < 0.001, d = −1.196) and step time (−) (*p* < 0.001, d = −0.615) also demonstrated meaningful group differences (Table 4).

### 3.3. Result of Feature Importance Analysis

**Figure 4:**
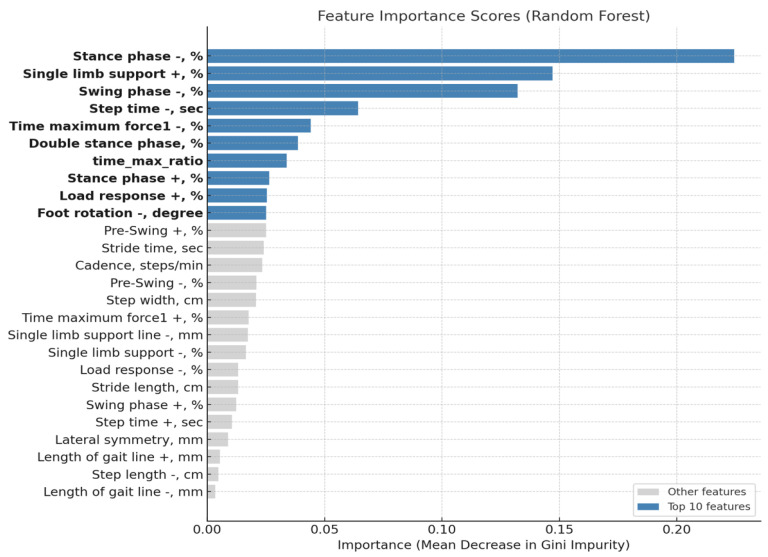
Feature importance scores by GINI Impurity of RF.

After excluding four features influenced by age (e.g., velocity, single-limb support line (+), foot rotation (+), step length (+)), the top 10 features with the highest importance scores were selected, and their relative contributions are visualized in Figure 4.

The most influential feature was stance phase (−) (Gini importance = 0.224), followed by single-limb support (+) (0.147), swing phase (−) (0.132), and step time (−) (0.064). Additional key features included time maximum force1 (−) (0.044), double stance phase (0.039), time_max_ratio (0.034), stance phase (+) (0.026), load response (+) (0.025), foot rotation (−), degree (0.025).

These top 10 features were subsequently used for feature-reduced model training, resulting in comparable or improved classification performance compared to models trained on the full feature set.

### 3.4. Result of Machine Learning Model

To evaluate classification performance, each model was trained and validated using both the full feature set (26 features) and a reduced feature set comprising the top 10 most important features selected via Random Forest. Table 5 summarizes the results of 5-fold stratified cross-validation across all models.

When using the full feature set, the Support Vector Machine (SVM) with a radial basis function kernel demonstrated the highest classification performance, achieving an accuracy of 93.8%, F1-score of 0.941, and a perfect ROC AUC of 1.000. Logistic Regression also performed robustly on the full dataset, with an accuracy of 91.1% and ROC AUC of 0.932, while Random Forest yielded slightly lower accuracy (87.5%) but a comparably high AUC (0.961).

**Figure 5:**
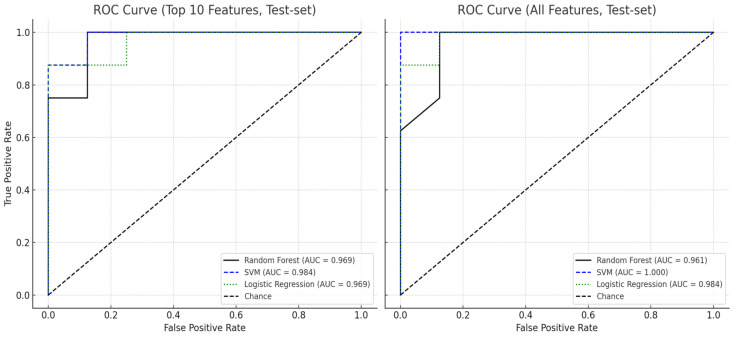
ROC curve comparison of three classifiers using top 10 and all features.

Upon restricting the input to the top 10 features, classification performance improved or remained stable across all models. Random Forest showed improved accuracy (92.3%) and F1-score (0.924), with a slight decrease in AUC to 0.949. The reduced-feature SVM achieved an accuracy of 91.1%, F1-score of 0.913, and maintained a high AUC of 0.961. Logistic Regression, using the same reduced features, also performed consistently well, with an accuracy of 91.1%, F1-score of 0.907, and AUC of 0.941 (Figure 5).

These results indicate that reducing the feature space to the most informative features not only maintained but, in some cases, enhanced model performance. Furthermore, while SVM demonstrated the highest discriminative capacity, both Random Forest and Logistic Regression achieved comparable results with the added benefit of interpretability and ease of integration in clinical settings.

### 3.5. Result of Machine Learning Model Using Non-Significant Variables

**Figure 6:**
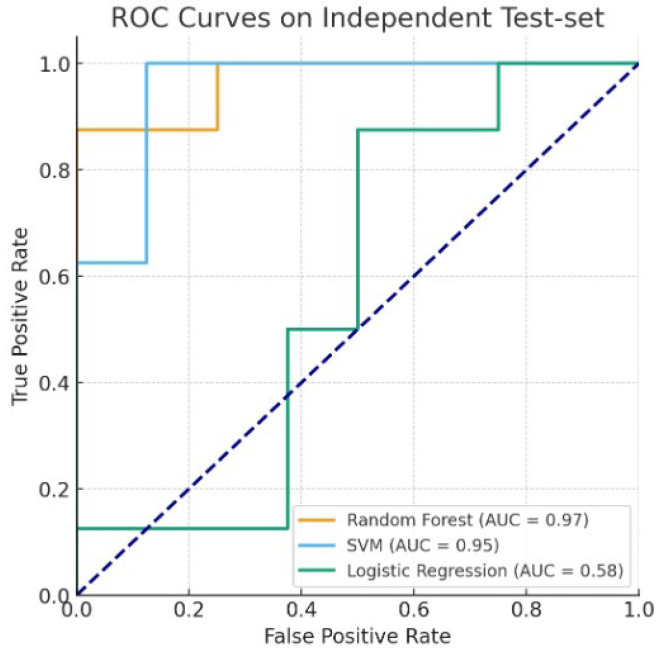
ROC curve comparison of three classifiers using non-significant (independent) features.

To examine whether the classification performance was solely driven by variables with significant group differences, we performed an additional analysis using only variables with *p* > 0.05 between the hemiplegic and mimicked hemiplegic gait groups. The variables included in this analysis were step length –, step width, step time +, stride time, velocity, single-limb support line +, single-limb support line –, lateral symmetry, and time maximum force1 +. Three classifiers—Random Forest, Support Vector Machine (SVM), and Logistic Regression classifiers—were trained on the training set (80%) and evaluated on the independent test set (20%) using Accuracy, F1-score, and ROC AUC metrics. The results are presented in Table 6 and Figure 6.

## 4. Discussion

This study aimed to identify key gait features and develop machine learning models to distinguish true hemiplegic gait (HG) from mimicked hemiplegic gait (MHG) using quantitative gait analysis data.

Gait features that can be collected through gait analysis include not only spatiotemporal features but also a wide variety of features related to the force, or movement of the center of pressure, making analysis difficult. In this study, machine learning was used to compare gait features obtained from numerous subjects in two groups to identify and classify the relationships between them. In this study, the collected gait analysis data were analyzed through machine learning, and the following results were obtained.

Firstly, In this study, ANCOVA revealed that four gait features—velocity, single-limb support line (+), foot rotation (+), and step length (+)—were significantly influenced by age. As a result, these features were excluded from intergroup statistical comparisons and machine learning modeling. Although each of these features represents clinically and biomechanically meaningful aspects of gait—including overall function, stability, joint control, and step efficiency—their strong association with age-related changes makes them less suitable for isolating pathological gait characteristics.

For example, velocity, a widely used marker of mobility and gait efficiency, is known to decrease with age due to reduced strength, balance, and neuromuscular coordination. Thus, differences in velocity may be attributed more to natural aging than to hemiparetic impairment [24,25]. Likewise, single-limb support line (+), derived from the trajectory of the center of pressure (COP), reflects the stability and duration of stance [26,27]. This feature is inherently sensitive to age-related decline in postural control, potentially confounding its interpretation as a group-specific gait deviation [28].

Foot rotation (+) indicates the degree of forefoot external rotation during gait. While such rotation is often seen in stroke-related compensatory movements, similar patterns can also emerge due to joint stiffness and muscular adaptations associated with aging, especially at the hip and ankle joints [29]. Step length (+), closely tied to stride mechanics, also decreases with age, as older adults typically exhibit reduced propulsion and joint range of motion. In this study, the absence of a significant difference in step length after adjusting for age supports this interpretation [30].

Importantly, all age-dependent gait variables identified in this study were derived from the unaffected side. While the unaffected side may exhibit more biomechanically stable patterns, it primarily reflects compensatory mechanisms rather than direct neurological impairments [31,32]. In contrast, the affected side provides richer pathological information, including asymmetry, altered timing, and deficits in force generation—features that are essential for distinguishing true hemiplegic gait from mimicked patterns [33]. Healthy individuals attempting to imitate hemiplegic gait are generally unable to replicate these subtle dysfunctions, making affected-side features especially valuable for classification. From a machine learning perspective, using predominantly affected-side parameters enhances discriminative power in differentiating neurologically impaired gait from cognitively generated imitation.

Secondly, a machine learning-based classification model was developed using unaffected-side gait features that were not significantly influenced by age. Feature importance analysis identified the top 10 features that most contributed to model performance. These features primarily comprised temporal gait features, phase-specific time ratios, and force timing characteristics, all of which captured essential distinctions between HG and MHG group.

Notably, features such as stance phase (−), single-limb support (+), swing phase (−), and step time (−) ranked among the most important. Among the top-ranked features identified in this study, stance phase (−), single-limb support (+), swing phase (−), and step time (−) reflect key spatiotemporal characteristics of hemiplegic gait. A reduced stance phase on the affected side indicates instability and diminished weight-bearing capacity, while increased single-limb support on the unaffected side reflects compensatory load-bearing strategies [31]. Shortened or delayed swing phase is commonly associated with impaired motor control and muscle weakness, and irregularities in step time reflect disrupted gait rhythm and temporal asymmetry [33]. These features capture pathological aspects of gait that are difficult for healthy individuals to replicate through mimicked hemiplegic gait, thus providing high discriminative power for classification. Although Rezgui et al. (2013) investigated the imitation of cerebral palsy gait rather than stroke-related hemiplegic gait, their findings support the notion that pathological gait patterns exhibit neuromechanical complexities that are difficult for healthy individuals to replicate accurately, thereby providing a relevant basis for our interpretation [34]. Their consistent emergence as important predictors in machine learning models highlights both their clinical relevance and their robustness in differentiating true hemiplegic gait from cognitively generated imitation. Furthermore, time-based kinetic features such as time_max_ratio and load response timing emerged as critical indicators. These features represent subtle shifts in the timing of ground reaction forces and suggest that temporal irregularities in neuromuscular response may serve as objective markers of pathological gait. Such micro-level deviations are unlikely to be perceived through visual inspection alone, underscoring the potential utility of data-driven metrics in clinical gait assessment.

Interestingly, the selected top features align well with those highlighted in previous clinical gait studies involving stroke patients. This convergence lends further support to their validity and highlights the importance of kinetic over kinematic features in distinguishing pathological gait. While kinematic features such as joint angles or step speed can be consciously manipulated by mimicry, kinetic features typically reflect involuntary motor output, offering greater diagnostic specificity.

In summary, the top 10 features identified in this study appear to capture fundamental biomechanical markers of true hemiplegic gait that are resistant to voluntary imitation. These findings suggest that carefully selected unaffected-side features—particularly those capturing temporal dynamics and weight transfer mechanisms—may serve as reliable components in objective gait classification systems. The results further support the use of explainable machine learning approaches to extract clinically relevant insights from quantitative gait analysis.

Thirdly, All three machine learning classification models developed in this study—Random Forest, Support Vector Machine (SVM), and Logistic Regression—demonstrated high performance in distinguishing hemiplegic gait (HG) from mimicked hemiplegic gait (MHG), supporting the feasibility of quantitative, data-driven gait classification. Among them, the SVM exhibited the highest discriminative power, achieving a perfect AUC (1.000) when trained on the full feature set and maintaining robust performance even when using only the top 10 features. This suggests that the SVM’s ability to capture nonlinear decision boundaries may be particularly well-suited for modeling the complex patterns observed in hemiplegic gait.

The Random Forest model also achieved high accuracy and provided the added benefit of interpretability through feature importance scores. These importance scores were used to guide dimensionality reduction and contributed to maintaining model performance with fewer features. Logistic Regression, although slightly lower in AUC compared to the other models, offers advantages in transparency and clinical interpretability. In clinical settings where decision-making must be explainable, such simplicity is often valued despite minor performance trade-offs.

Importantly, models trained on only the top 10 most important features yielded comparable performance to models trained on the full feature set. This highlights the potential for efficient and lightweight gait assessment systems that minimize data collection burden without sacrificing diagnostic power. Such models may be more easily deployed in real-world settings, including wearable devices or bedside evaluation tools, and may support functional evaluations for rehabilitation planning, insurance verification, and return-to-work decisions. these models provide clinically meaningful insights into gait asymmetry and compensatory strategies. For example, the observed alterations in stance and swing phases correspond to well-known biomechanical consequences of muscle weakness and postural instability. Importantly, we found that models trained on only the top 10 most important features yielded comparable performance to those trained on the full feature set. This highlights the feasibility of developing efficient and lightweight gait assessment systems that minimize data collection burden without compromising diagnostic power. Such systems could be particularly advantageous for deployment in real-world clinical environments, including wearable devices or bedside evaluation tools, to support functional assessments for rehabilitation planning, insurance verification, and return-to-work decisions. Moreover, it should be acknowledged that the laterality of motor deficits may exert a significant influence on gait characteristics. Patients with left-sided hemiparesis often present with spatial neglect, which can exacerbate asymmetry and impair motor control, whereas right-sided hemiparesis may follow distinct compensatory mechanisms. Although our dataset did not allow for stratified analysis by side of involvement, future studies with larger cohorts should investigate laterality-specific gait alterations and their implications for personalized rehabilitation strategies. In addition, external validation using larger, multi-center datasets will be crucial to establish the robustness and generalizability of such lightweight models. Future research should also explore the integration of advanced algorithms, including deep learning and ensemble methods, to further enhance predictive performance while maintaining clinical interpretability.

Even when the analysis was restricted to variables showing no significant differences between groups (*p* > 0.05), Random Forest and SVM maintained high classification performance (ROC AUC = 0.97 and 0.95, respectively; Accuracy = 87.5%), demonstrating that the models learned complex multivariate patterns rather than exploiting obvious contrasts between groups. In contrast, Logistic Regression achieved substantially lower performance (ROC AUC = 0.58; Accuracy = 62.5%). This discrepancy can be attributed to the linear nature of Logistic Regression, which cannot adequately capture nonlinear relationships and higher-order interactions likely present in the selected variables. Moreover, potential multicollinearity among gait features and the limited sample size may have further reduced its performance. These findings collectively highlight the importance of nonlinear classification models in capturing complex gait patterns, particularly when apparent group differences are removed.

Several limitations of the present study should be acknowledged.

First, the training methodology for the MHG group poses concerns regarding ecological validity. Although participants were instructed using videos of actual stroke patients and demonstrations by doctors and were asked to reproduce characteristic hemiplegic gait patterns (hip extension and internal rotation, knee extension, ankle plantarflexion and inversion, hip hiking, and circumduction), no objective criteria or agreement scale was applied to confirm accurate imitation. Gait analysis was conducted only when physicians judged the mimicry to be sufficient, and treadmill speed was standardized at 0.7 km/h to minimize variability. Nevertheless, reliance on subjective assessment may have introduced uncontrolled variability, potentially compromising comparability with the HG group and leading to overestimation of the model’s discriminatory capacity.

Second, the relatively small sample size (N = 79) limited the statistical power of the study. While sensitivity analysis confirmed that medium-to-large effects could be reliably detected, smaller effects might have been overlooked. According to Cohen’s conventional thresholds for multiple regression (f^2^ = 0.02 for small, f^2^ = 0.15 for medium, and f^2^ = 0.35 for large effects), our calculated f^2^ = 0.2 corresponds to a medium-sized effect. This suggests that although the independent variables included in the model influence the dependent variable, the magnitude of this influence may not be substantial. Nevertheless, even very small effects can have considerable practical or clinical importance if they affect a large population.

Third, the machine learning models were trained and tested on a single-center dataset, raising concerns about external validity. To mitigate this, we applied cross-validation and developed a reduced model using only the top 10 key features identified through feature importance analysis, which showed comparable performance to the full model. However, such approaches cannot fundamentally resolve the limitation. We are therefore preparing additional patient recruitment and multi-center collaborative studies, and future work will focus on external validation with larger datasets to strengthen the generalizability of the model.

Fourth, although our models demonstrated high classification accuracy, it must be acknowledged that several gait features exhibited significant group differences between HG and MHG. This may have biased the classification results, favoring automatic discrimination and potentially inflating model performance beyond what might be expected in more ecologically valid conditions. Lastly, while we employed three widely used algorithms (Random Forest, SVM, Logistic Regression), future studies may benefit from exploring deep learning architectures, ensemble approaches, and refined threshold-tuning strategies to further enhance predictive accuracy and clinical applicability.

## 5. Conclusions

This study demonstrated that machine learning models can effectively distinguish hemiplegic gait from mimicked hemiplegic gait using quantitative gait features. By applying Random Forest, Support Vector Machine (SVM), and Logistic Regression classifiers, high classification performance was achieved even with a limited dataset, with SVM showing the highest AUC values. Feature importance analysis revealed that temporal asymmetries, such as altered stance and swing phases, along with force timing differences and spatial instability, were key discriminators. Notably, models trained using only the top 10 most informative features achieved comparable performance to those using the full feature set, suggesting that lightweight and interpretable models may be sufficient for clinical application. These findings support the potential of machine learning-based gait analysis as an objective tool for identifying pathological gait characteristics and contribute to the development of data-driven neurorehabilitation strategies. Further validation using external datasets and integration of additional physiological signals is recommended to enhance clinical utility and generalizability.

## Figures and Tables

**Table 1:** Inclusion and exclusion criteria for the HG and MHG group.

	Inclusion Criteria	Exclusion Criteria
HG group	- Individuals diagnosed with stroke via CT or MRI and who have hemiparesis	- Individuals with clinical symptoms that could affect their walking ability (such as musculoskeletal diseases, acute sprain, etc.)
	- Individuals capable of independent walking for more than 30 s on a treadmill (with Manual Muscle Testing (MMT) grades 3–5 for lower limbs, and Functional Ambulation Categories (FAC) 9 level 3–5).	
MHG group	- Individuals who have not been diagnosed with stroke	
	- Individuals who can mimic hemiplegic gait following the instructions of the medical staff	

**Table 2:** Demographic characteristics of subjects.

Characteristics		HG Group	MHG Group
Subjects (number)		39	40
Sex (number (%))	Male	20 (51.2%)	28 (70%)
	Female	19 (48.8%)	12 (30%)
Age (Mean (SD))		62.8 (6.44)	26.98 (2.27)
Affected side (number (%))	Left	19 (47.5%)	20 (50%)
	Right	21 (52.5%)	20 (50%)
MMT (median (IQR))	Upper limb	4 (1)	5 (0)
	Lower limb	4 (0)	5 (0)

HG, hemiplegic gait; MHG, mimicked hemiplegic gait; SD, standard deviation; IQR, interquartile range (at least 50% of the observations share the same value); MMT, manual muscle test.

**Table 3:** Gait features obtained by treadmill.

	Feature (Unit)	Description
Spatial feature	Foot rotation (degree)	positive: external rotation/negative: internal rotation
	Step length (cm)	from heel contact of one foot to the other foot
	Stride length (cm)	initial contact of the same foot
	Step width (cm)	width between the feet
Temporal feature	Step time (sec)	step time is the time taken between the heel contact of one foot and the heel contact of the other foot
	Stride time (sec)	stride time is the elapsed time between the first contact of two consecutive footprints of the same foot
	Cadence (steps/min)	steps per minute
	Velocity (km/h)	walking speed during gait analysis
CoP feature	Length of gait line (mm)	CoP movement on one foot during the entire stance phase
	Single-limb support line (mm)	CoP movement during the single-leg support
	Lateral symmetry (mm)	horizontal distance from the center point of the horizontal line
Gait event Feature	Stance phase (%)	from heel strike to toe off
	Load response (%)	begins with initial contact, the instant the foot contacts the ground
	Single-limb support (%)	the swing phase where only one limb in in contact with the ground
	Pre-swing (%)	final phase of stance, starting with initial contact of the opposite limb
	Swing phase (%)	period during which the foot is in the air
	Double stance phase (%)	both feet are simultaneously in contact with the ground
Force feature	Time maximum force 1 (%)	first maximum vertical force, which occurs at the end of loading response

**Table 4:** Comparison of features.

Features (Test Type)	HG Group	MHG Group	*p*-Value	Cohen’s d	Gini
#Foot rotation (+) (b)	10.74 ± 4.97	3.43 ± 4.82	0.000 **	−1.492	0.051
Foot rotation (−) (b)	13.63 ± 8.14	3.96 ± 10.05	0.000 **	−1.056	0.037
#Step length (+) (b)	17.48 ± 8.73	23.19 ± 7.02	0.002 *	0.723	0.019
Step length (−) (b)	19.64 ± 8.48	20.32 ± 7.58	0.709	−0.084	0.004
Stride length (a)	37.12 ± 15.64	43.51 ± 6.34	0.022 *	−0.538	0.011
Step width (a)	15.27 ± 3.51	16.37 ± 4.49	0.230	−0.272	0.012
Stance phase (+) (a)	74.10 ± 5.54	78.32 ± 5.15	0.001 **	−0.791	0.018
Stance phase (−) (b)	72.74 ± 5.37	59.00 ± 7.50	0.000 **	2.103	0.138
Load Response (+) (b)	23.59 ± 5.11	19.48 ± 6.31	0.002 *	0.715	0.018
Load Response (−) (a)	23.72 ± 4.71	17.80 ± 4.71	0.000 **	1.256	0.038
Single-limb support (+) (b)	26.98 ± 5.61	41.06 ± 7.56	0.000 **	−2.110	0.144
Single-limb support (−) (b)	25.26 ± 6.31	21.67 ± 5.13	0.007 *	0.625	0.011
Pre-Swing (+) (a)	23.55 ± 4.93	17.79 ± 4.71	0.000 **	1.195	0.027
Pre-Swing (−) (b)	23.61 ± 5.29	19.50 ± 6.32	0.002 *	0.704	0.019
Swing phase (+) (a)	25.90 ± 5.54	21.68 ± 5.15	0.001 **	0.791	0.014
Swing phase (−) (b)	27.26 ± 5.37	41.00 ± 7.50	0.000 **	−2.103	0.155
Double stance phase (a)	47.10 ± 8.36	37.23 ± 8.09	0.000 **	1.200	0.037
Step time (+) (b)	0.98 ± 0.58	0.87 ± 0.22	0.274	0.252	0.008
Step time (−) (b)	1.02 ± 0.67	1.33 ± 0.22	0.010 *	−0.615	0.061
Stride time (b)	2.00 ± 1.24	2.20 ± 0.31	0.346	−0.217	0.016
Cadence (a)	72.66 ± 25.57	55.78 ± 8.37	0.000 **	0.892	0.017
#Velocity (a)	0.75 ± 0.32	0.71 ± 0.01	0.501	−0.155	0.025
Length of gait line (+) (a)	124.13 ± 22.62	134.88 ± 24.10	0.044 *	−0.460	0.007
Length of gait line (−) (a)	115.44 ± 27.07	101.73 ± 17.74	0.010 *	0.601	0.007
#Single-limb support line (+) (b)	36.55 ± 20.92	42.19 ± 14.65	0.171	0.313	0.005
Single-limb support line (−) (b)	22.26 ± 15.07	24.47 ± 8.32	0.424	−0.182	0.011
Lateral symmetry (a)	−14.51 ± 24.26	−21.60 ± 18.50	0.149	0.329	0.002
Time maximum force 1 (+) (b)	26.74 ± 5.44	28.50 ± 8.25	0.267	−0.251	0.013
Time maximum force 1 (−) (a)	28.67 ± 5.25	20.52 ± 5.22	0.000 **	1.556	0.042
Time maximum force ratio (b)	0.95 ± 0.19	1.46 ± 0.57	0.000 **	−1.196	0.035

Data are presented as mean ± standard deviation (SD). HG, hemiplegic gait; MHG, mimicked hemiplegic gait. (a): independent *t*-test; (b): Mann–Whitney U test. * *p*-value < 0.05; ** *p* value < 0.001; #: features excluded due to age. For HG and MHG groups, the values of the unaffected side and affected side were entered as (+) and (−), respectively. In case of the reference value, values corresponding to the right and left sides were entered in (+) and (−), respectively.

**Table 5:** The test results of classification models using top 10 features.

	Feature Set	Accuracy	F-1-Score	ROC AUC
Random Forest	All features	0.875	0.889	0.961
	Top 10 features	0.923	0.924	0.949
SVM (RBF Kernel)	All features	0.938	0.941	1.000
	Top 10 features	0.911	0.913	0.961
Logistic Regression	All features	0.911	0.901	0.932
	Top 10 features	0.911	0.907	0.941

**Table 6:** The test results of classification models using non-significant features.

	Accuracy	F-1-Score	ROC AUC
Random Forest	0.875	0.875	0.969
SVM (RBF Kernel)	0.875	0.889	0.953
Logistic Regression	0.625	0.667	0.578

## Data Availability

The data presented in this study are available upon request from the corresponding author. These data are not publicly available because of privacy concerns.
